# Dual role of PpV in *Drosophila* crystal cell proliferation and survival

**DOI:** 10.1093/jmcb/mjae028

**Published:** 2024-07-31

**Authors:** Wang Luo, Fang Zhang, Fangzhen Zhao, Yang Fang, Long Zhao, Ying Su

**Affiliations:** Key Laboratory of Evolution & Marine Biodiversity (Ministry of Education) and Institute of Evolution & Marine Biodiversity, Ocean University of China, Qingdao 266003, China; College of Marine Life Sciences, Ocean University of China, Qingdao 266003, China; Key Laboratory of Evolution & Marine Biodiversity (Ministry of Education) and Institute of Evolution & Marine Biodiversity, Ocean University of China, Qingdao 266003, China; College of Marine Life Sciences, Ocean University of China, Qingdao 266003, China; Key Laboratory of Evolution & Marine Biodiversity (Ministry of Education) and Institute of Evolution & Marine Biodiversity, Ocean University of China, Qingdao 266003, China; College of Marine Life Sciences, Ocean University of China, Qingdao 266003, China; Key Laboratory of Evolution & Marine Biodiversity (Ministry of Education) and Institute of Evolution & Marine Biodiversity, Ocean University of China, Qingdao 266003, China; College of Marine Life Sciences, Ocean University of China, Qingdao 266003, China; Key Laboratory of Evolution & Marine Biodiversity (Ministry of Education) and Institute of Evolution & Marine Biodiversity, Ocean University of China, Qingdao 266003, China; Fisheries College, Ocean University of China, Qingdao 266003, China; Key Laboratory of Evolution & Marine Biodiversity (Ministry of Education) and Institute of Evolution & Marine Biodiversity, Ocean University of China, Qingdao 266003, China; College of Marine Life Sciences, Ocean University of China, Qingdao 266003, China

**Keywords:** protein phosphatase, blood cells, Notch pathway, lymph gland, mechanical injury

## Abstract

*Drosophila melanogaster* crystal cells are a specialized type of blood cells for the innate immune process upon injury. Under normal conditions, crystal cells rarely proliferate and constitute a small proportion of fly blood cells. Notch signaling has been known to guide the cell fate determination of crystal cells and maintain their survival. Here, we reported that protein phosphatase V (PpV), the unique catalytic subunit of protein phosphatase 6 in *Drosophila*, is a novel regulator of crystal cell proliferation and integrity. We found that PpV proteins highly accumulated in crystal cells in the larval hematopoietic organ termed the lymph gland. Silencing *PpV* using RNA interference led to increased crystal cell proliferation in a Notch-independent manner and induced crystal cell rupture dependent on Notch signaling. Moreover, additive PpV prevented the rupture of crystal cells in lymph glands upon a needle injury, suggesting the involvement of PpV in wound healing. Altogether, our results indicated that PpV plays a dual role in lymph glands, preventing crystal cell proliferation to limit the cell number, as well as inhibiting crystal cell rupture to maintain their survival.

## Introduction

Protein phosphatases are well-known to reverse the protein phosphorylation process, antagonizing the action of protein kinases. They have diverse structures, substrate selections, and physiological functions as well (Shi, 2009; Brautigan, 2013; Zhao et al., 2017; Ruvolo, 2019; Zhang et al., 2023). *Drosophila* gene *protein phosphatase V* (*PpV*) encodes the catalytic subunit of protein phosphatase 6 (PP6), an evolutionarily conserved protein serine/threonine phosphatase from yeast to human (Liu et al., 2019, 2021). The functions of PP6 have been studied in several processes, such as mitosis, lymphocyte development, and cancer (Zhong et al., 2011; Hammond et al., 2013; Kato et al., 2015; Ye et al., 2015; Ertych et al., 2016; Ziembik et al., 2017). The mutation of PPP6C (catalytic subunit of PP6 in mammals) at a conserved histidine within the catalytic site is often found in human malignant melanoma (Hammond et al., 2013). The dysfunction of PP6 can decrease its catalytic activity toward the substrate, the mitotic kinase Aurora A, thereby causing the abnormal form of spindle and DNA damage during mitosis (Zeng et al., 2010; Hodis et al., 2012; Hammond et al., 2013). PP6 was also reported to regulate T-cell development in mice (Ye et al., 2015). Based on this limited number of reports, the physiological functions of PP6 are far away from being well understood.

Previous studies by our group and colleagues demonstrated that PpV regulates wing development through Hedgehog and JNK signaling in *Drosophila* (Chi et al., 2018; Liu et al., 2021). *PpV* was also reported to modulate *Drosophila* oogenesis and early embryogenesis (Liu et al., 2019), lipid homeostasis (Yin et al., 2014), tumor growth and invasion (Ma et al., 2017). To explore the role of PpV in other organs/tissues, we knocked down *PpV* expression in whole larvae using RNA interference (RNAi) and conducted RNA sequencing (RNA-seq) analysis. The RNA-seq data revealed that the expression levels of many hematopoiesis-related genes were changed in *PpV RNAi* larvae. However, the relationship between PpV and hematopoiesis is still unclear.

In *Drosophila*, there are three major types of blood cells, plasmatocytes, crystal cells, and lamellocytes, which are homologous to the mammalian myeloid blood cells and essential for innate immune responses (Jung et al., 2005). Plasmatocytes function as macrophages in the *Drosophila* immune system, eliminating microbial pathogens and dead cells (Jung et al., 2005; Yu et al., 2018). Crystal cells are required for melanization, an insect-specific immune process (Waltzer et al., 2003). During injury or wasp parasitic stress, crystal cells burst themselves to release prophenoloxidases (PPOs), which catalyze phenols into quinones to participate in the melanization process (Cerenius and Soderhall, 2004). Lamellocytes are largely produced in response to wasp parasitization, encapsulating the invading wasp eggs to undergo further removal (Rizki and Rizki, 1991). Fly hemocytes are produced from two waves of hematopoietic processes at the embryonic stage and the larval stage (Coates et al., 2021). To delineate the physiological function of PpV in larval hematopoiesis, we focused on the lymph gland, the specialized hematopoietic organ in the larval stage (Krzemien et al., 2010; Morin-Poulard et al., 2021; Luo et al., 2022), and investigate the novel function of PpV in the maintenance of the crystal cell population by depletion or overexpression of *PpV* in crystal cells.

## Results

### Systemic knockdown of PpV in *Drosophila* larvae causes increased expression of hemocyte-related genes

**Figure 1:**
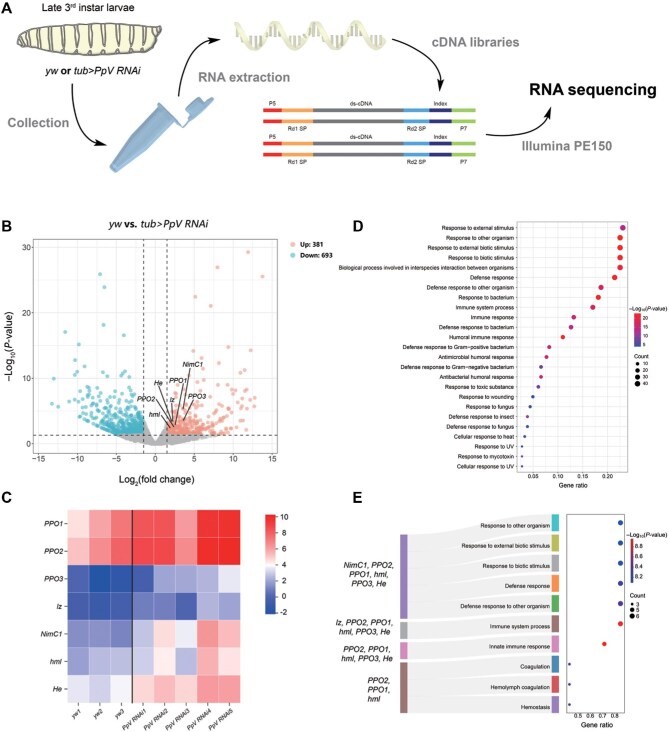
The transcriptome *of tub>PpV RNAi* larvae vs. *yw* larvae. (**A**) Schematic diagram of RNA-seq. (**B**) Volcano plot of DEGs upon *PpV* knockdown. The X-axis represents the change of gene expression level between *tub>PpV RNAi* and *yw* larvae. The Y-axis represents the significance level of gene expression difference. The up-regulated genes are indicated by red dots (*P* < 0.05), the down-regulated genes are indicated by blue dots (*P* < 0.05), and the genes that have no significant changes are indicated by grey dots. (**C**) Heatmap showing differential expression of hemocyte marker genes. The X-axis represents individual samples, and the Y-axis represents hemocyte marker genes. The expression level gradually increases from blue to red. (**D** and **E**) GO analysis of all DEGs (**D**) and differentially expressed hemocyte marker genes (**E**). The Y-axis represents GO terms, and the X-axis represents the ratio of significant DEGs in the corresponding term to all genes in the term. Dot size represents the number of genes enriched in the corresponding term. Dot color represents the significance level of enrichment, gradually increasing from blue to red.

To establish the transcriptome of wild-type larvae (*yw*) and systemic *PpV* knockdown larvae (*tub-gal4* was crossed with *UAS-PpV RNAi*; in short as *tub>PpV RNAi*), we collected the 3rd instar larvae with specific genotypes to conduct RNA-seq (Figure 1A). By analyzing the RNA-seq data, 1074 differentially expressed genes (DEGs) upon *PpV* knockdown were identified, including 381 up-regulated genes and 693 down-regulated genes (Figure 1B). In particular, the expression levels of many hemocyte marker genes, including the pan-hemocyte marker gene *Hemese* (*He*), plasmatocyte markers *hemolectin* (*hml*) and *Nimrod C1* (*NimC1*), crystal cell markers *lozenge* (*lz*) and prophenoloxidase family members *PPO1, PPO2*, and *PPO3*, were all significantly increased upon the knockdown of *PpV* in whole larvae (Figure 1B and C; Lebestky et al., 2003; Waltzer et al., 2003; Jung et al., 2005; Ferjoux et al., 2007; Banerjee et al., 2019; Cattenoz et al., 2021; Rodrigues et al., 2021). In agreement, Gene Ontology (GO) analysis of the DEGs revealed that *PpV* knockdown mainly affected the pathways related to immune responses (Figure 1D and E), as hemocytes in *Drosophila* execute the immune defense. Collectively, the transcriptomic data suggested that PpV may play a role in the hematopoietic system in *Drosophila* larvae.

### PpV is highly expressed in crystal cells in the lymph gland

**Figure 2:**
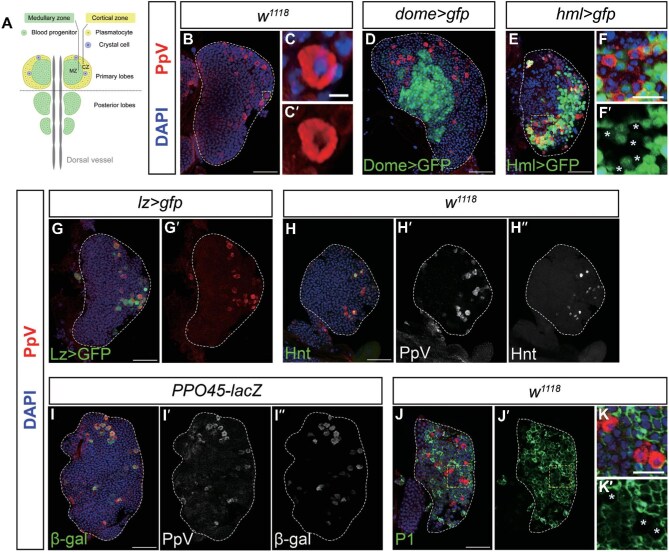
PpV is highly expressed in crystal cells. (**A**) Schematic diagram of the lymph gland in late 3rd instar larvae. (**B**–**C′**) PpV (red) is highly expressed in a small part of cells in the 3rd instar primary lobes of wild-type (*w^1118^*) larvae (**B**) and accumulated in the cytoplasm of these PpV^high^ cells (**C** and **C′**, 8× enlarged view of the white-dashed box in **B**). (**D**) PpV^high^ cells (red) do not overlap with blood progenitors (Dome>GFP) in the MZ. (**E**–**F′**) PpV^high^ cells (red) are located in the CZ (**E**) but some of them do not overlap (white asterisks) with mature blood cells (Hml>GFP) in the CZ (**F** and **F′**, 3.2× enlarged view of the white-dashed box in **E**). (**G** and **G′**) PpV^high^ cells (red) highly overlap with crystal cells (Lz>GFP) in the CZ. (**H**–**H″**) PpV^high^ cells (red) are highly superposed with the crystal cell marker Hnt (green) in *w^1118^* larvae. (**I**–**I″**) PpV^high^ cells (red) are highly superposed with β-gal staining (green), which labels mature crystal cells, in *PPO45-lacZ* larvae. (**J**–**K′**) PpV^high^ cells (red) are not P1-marked plasmatocytes (green) in *w^1118^* larvae (white asterisks). Panels **K** and **K′** are the 3.2× enlarged view of white-dashed boxes in **J**, and **J′**, respectively. DAPI (blue) marks nuclei. Scale bar, 50 μm, except 5 μm for **C** and **C′** or 20 μm for **F, F′, K**, and **K′**.

We then explored the function of PpV in *Drosophila* hematopoietic organ, the lymph gland. In late 3rd instar larvae, the fully mature lymph gland consists of 3–4 pairs of lobes aligned symmetrically along the dorsal vessel (Grigorian et al., 2011). The anterior pair of lobes, called the primary lobes, is the biggest and comprises both blood progenitors in the medial medullary zone (MZ) and differentiated blood cells, such as plasmatocytes and crystal cells, in the lateral cortical zone (CZ) (Figure 2A; Grigorian et al., 2011; Mihajlovic et al., 2019).

First, we used a specific antibody to examine the expression pattern of PpV protein in late 3rd instar lymph glands. As shown in Figure 2B–C′, in wild-type (*w^1118^*) larvae, PpV was expressed at low levels in the primary lobes of the lymph gland and specifically accumulated in the cytoplasm of a small group of cells (PpV^high^ cells). To clarify the cell type of the PpV^high^ population, we used two well-recognized drivers, *dome-gal4,UAS-gfp* (*dome>gfp* in short for genotype, Dome>GFP for protein) and *hml-gal4,UAS-gfp* (*hml>gfp*), to outline the MZ and CZ compartments, respectively (Banerjee et al., 2019). PpV^high^ cells were located outside the Dome>GFP-indicated MZ region (Figure 2D) but within the Hml>GFP-outlined CZ region (Figure 2E), suggesting that they could be differentiated blood cells but not progenitor cells. Under normal conditions, two major types of differentiated hemocytes, plasmatocytes and crystal cells, reside in the CZ region of lymph glands. Plasmatocytes continuously express *hml*, from differentiating cells to fully mature cells. In contrast, crystal cells discontinue the expression of *hml* when fully mature (Mukherjee et al., 2011). Not all PpV^high^ cells were labeled by Hml>GFP (Figure 2F and F′), implying that they might belong to the lineage of crystal cells.

Then, we used *lz-gal4,UAS-gfp* (*lz>gfp*) to label the whole lineage of crystal cells from differentiation initiation to complete maturity. PpV staining showed that PpV^high^ cells were highly superposed with Lz>GFP-labeled (Lz^+^) cells (Figure 2G and G′). By co-staining with the anti-Hnt antibody in *w^1118^* or β-gal in *PPO45-lacZ* line that indicates mature crystal cells (Ferjoux et al., 2007), PpV signals co-localized well with Hnt or β-gal signals (Figure 2H–I′′). On the other hand, the staining signal against the P1 antigen, indicating mature plasmatocytes, did not overlap with the PpV signal (Figure 2J–K′). In addition, we analyzed the previously published single-cell transcriptomic data of *Drosophila* larval lymph gland (Cho et al., 2020), which revealed that the mean level of *PpV* expression is high in crystal cells but low in other hemocytes (Supplementary Figure S1A). These results together demonstrated that PpV is highly expressed in crystal cells in lymph glands.

### PpV is essential for the maintenance of crystal cell number and survival

**Figure 3:**
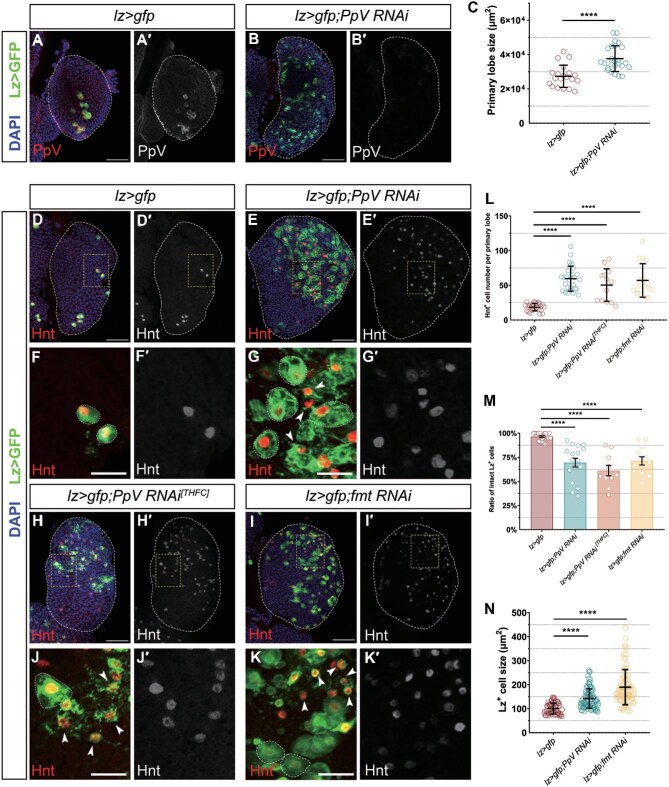
PpV regulates the number and integrity of crystal cells. (**A**–**B′**) PpV staining (red) is abrogated from the lymph gland upon *PpV RNAi*. (**C**) Quantification of the overall primary lobe size for genotypes shown. Error bars indicate SD; *n* = 20 and 25. (**D**–**K′**) Hnt staining of crystal cells in *lz>gfp, lz>gfp;PpV RNAi, lz>gfp;PpV RNAi^[THFC]^*, and *lz>gfp;fmt RNAi* lymph glands. Crystal cells (Lz>GFP) in *lz>gfp;PpV RNAi* (**G** and **J**, white arrows) and *lz>gfp;fmt RNAi* lymph glands (**K**, white arrows) are drastically ruptured compared to those in the *lz>gfp* control (**F**), while the Hnt-marked nuclei (red in **F, G, J**, and **K**; grey in **F′, G′, J′**, and **K′**) have no obvious change. Panels **F**–**G′** and **J**–**K′** are the 4× enlarged view of white-dashed boxes in **D**–**E′** and **H**–**I′**, respectively. (**L**) The number of Hnt^+^ crystal cells per primary lobe for genotypes shown. Error bars indicate SD; *n* = 26, 27, 13, and 16. (**M**) The proportion of intact Lz^+^ crystal cells for genotypes shown. Error bars indicate SEM; *n* = 21, 17, 10, and 12. (**N**) Quantification of the Lz^+^ crystal cell size for genotypes shown. Error bars indicate SD; *n* = 60, 100, and 80. DAPI (blue) marks nuclei. Scale bar, 50 μm (**A**–**B′, D**–**E′**, and **H**–**I′**) or 20 μm (**F**–**G′** and **J**–**K′**). ****P < 0.0001.

To determine whether PpV plays a unique role in crystal cell lineage, we knocked down the *PpV* expression, specifically in crystal cells, by expressing *PpV RNAi* under the control of *lz-gal4* (*lz>gfp;PpV RNAi*). As expected, PpV expression in the 3rd instar lymph gland was eliminated (Figure 3A–B′).

We noticed that the primary lobe size was mildly but significantly enlarged upon *PpV* depletion (Figure 3C). Furthermore, Hnt staining showed that the number of crystal cells in these *PpV RNAi* lymph glands dramatically increased compared to the *lz>gfp* control (Figure 3D–E′ and L). Meanwhile, the Lz>GFP signal revealed that many crystal cells (∼30% on average) in the *PpV RNAi* lymph glands were broken (Figure 3G and M). Cytoskeletal integrity of these ruptured crystal cells was damaged, as indicated by phalloidin staining (Supplementary Figure S2), while the nuclei were almost intact in these ruptured cells, shown by the Hnt signal (Figure 3G′). When another *PpV RNAi* strain was used, similar phenotypes were observed, including an increased number of crystal cells (Figure 3H and L) and crystal cell rupture (Figure 3J and M). Besides, the remaining intact crystal cells in the *PpV RNAi* lymph glands exhibited a larger size than normal (Figure 3N), possibly destined to break after further swelling.

Considering that PpV protein serves as the catalytic subunit of PP6 holoenzyme, we examined whether other subunits of the PP6 holoenzyme can affect crystal cells. Utilizing a transgenic RNAi line under the control of *lz-gal4*, we knocked down the expression of *fiery mountain* (*fmt*), which encodes the regulatory subunit of PP6 in *Drosophila*. Phenocopying *PpV* knockdown, *lz>gfp;fmt RNAi* lymph glands also showed higher number of crystal cells (Figure 3I and L), ∼30% crystal cell rupture (Figure 3K and M), and enlarged size of intact crystal cells (Figure 3N), suggesting that PpV may function through PP6 holoenzyme to regulate the number and integrity of crystal cells in lymph glands.

**Figure 4:**
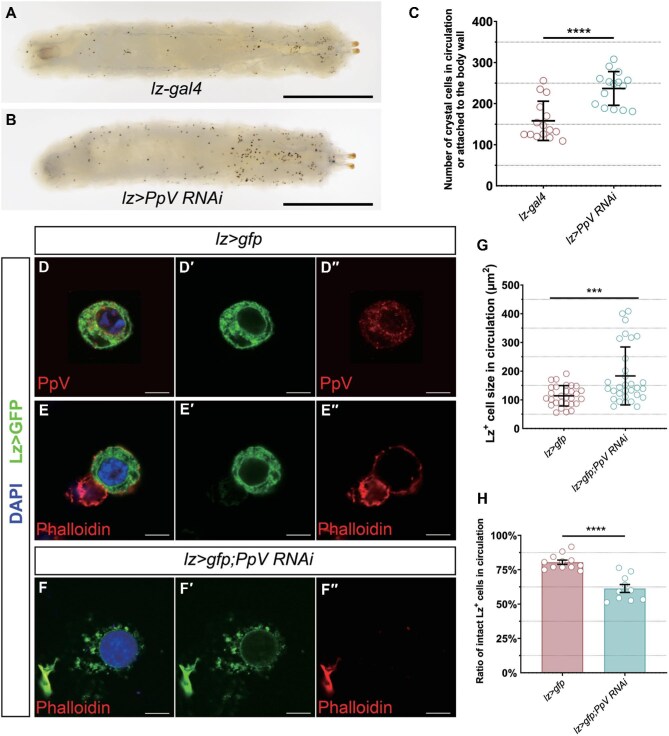
PpV regulates the number and integrity of crystal cells in circulation. (**A** and **B**) The crystal cells on larval body wall or in circulation increase upon *PpV* knockdown. Scale bar, 1 mm. (**C**) The number of crystal cells in circulation or attached to the body wall for genotypes shown. Error bars indicate SD; *n* = 15. (**D**–**F″**) PpV is expressed in the intact circulating crystal cells (Lz>GFP) in the *lz>gfp* control, while crystal cell rupture is observed in *lz>gfp;PpV RNAi* larvae. Phalloidin (red) indicates cytoskeleton. DAPI (blue) marks nuclei. Scale bar, 6 μm. (**G**) Quantification of the Lz^+^ cell size in circulation for genotypes shown. Error bars indicate SD; *n* = 30. (**H**) The proportion of intact Lz^+^ cells in circulation for genotypes shown. Error bars indicate SEM; *n* = 13 and 10. ***P < 0.001, ****P < 0.0001.

Since *Drosophila* hematopoiesis takes place in two temporally distinct waves at the embryonic stage and the larval stage, we then checked the expression pattern of PpV in circulating hemocytes, which are derived from the embryonic stage (Evans et al., 2022). By immunostaining, we demonstrated that the PpV protein was expressed in circulating crystal cells (Figure 4D–D″). We analyzed another single-cell RNA-seq data for circulating hemocytes and sessile blood cells (Tattikota et al., 2020), which indicated that the expression of *PpV* is concentrated in the crystal cell population with abundant PPO1 expression (PPO1^high^) (Supplementary Figure S1B). In order to determine whether PpV regulates circulating crystal cells, we heated the 3rd instar larvae to activate the PPO enzyme and visualize the crystal cells (black in color) (Bidla et al., 2007). Likewise, this part of crystal cells increased in number upon *PpV* knockdown (Figure 4A–C). *PpV* ablation in circulating crystal cells also resulted in plasma membrane disruption (Figure 4E–F′′ and H) and larger cell size (Figure 4G). Taken together, *PpV* depletion in crystal cells can affect their number, size, and integrity, suggesting a critical role of PpV in the maintenance of crystal cell homeostasis.

### Hml^+^ precursors require PpV to regulate the number of crystal cells

**Figure 5:**
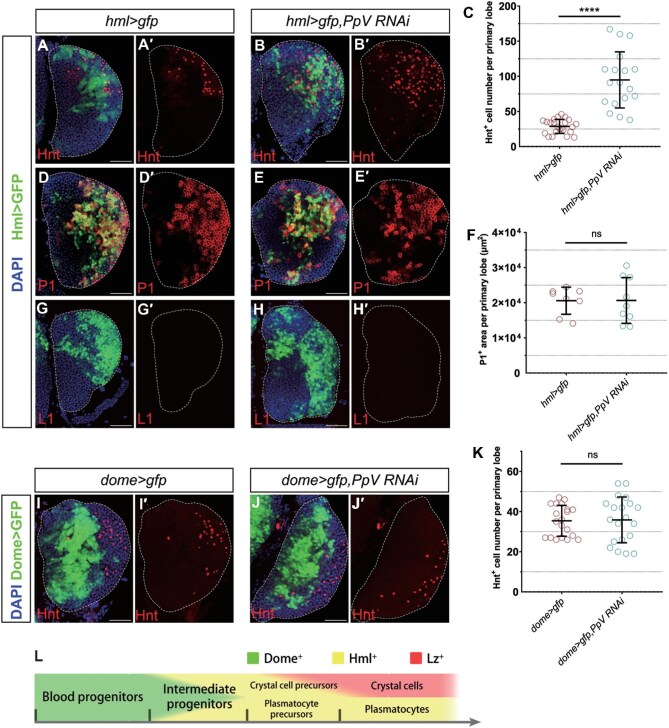
PpV regulates the number of crystal cells from the Hml^+^ precursor stage but has no effect on plasmatocytes. (**A**–**B′**) Hnt^+^ (red) crystal cells increase in the *hml>gfp,PpV RNAi* lymph gland, compared to the *hml>gfp* control. (**C**) The number of Hml^+^ crystal cells per primary lobe for genotypes shown. Error bars indicate SD; *n* = 23 and 18. (**D**–**E′**) P1-labeled plasmatocytes (red) have no difference in number between *hml>gfp* control and *hml>gfp,PpV RNAi* lymph glands. (**F**) The area of P1^+^ plasmatocytes for genotypes shown. Error bars indicate SD; *n* = 8 and 9. (**G**–**H′**) Knocking down *PpV* by RNAi does not generate lamellocytes (L1 staining in red). (**I**–**J′**) Hnt^+^ (red) crystal cells have no difference in number between *dome>gfp* control and *dome>gfp,PpV RNAi* lymph glands. (**K**) The number of Hnt^+^ crystal cells per primary lobe for genotypes shown. Error bars indicate SD; *n* = 20. (**L**) Schematic diagram of the development course of hemocytes in the lymph gland. GFP driven by *hml-gal4* (Hml>GFP) marks the CZ. GFP driven by *dome-gal4* (Dome>GFP) marks the MZ. DAPI (blue) marks nuclei. Scale bar, 50 μm. ****P < 0.0001; ns, no significance.

Hml has been reported as one of the earliest markers representing blood cell differentiation within the lymph gland (Evans et al., 2014). Crystal cells originate from Hml^+^ precursors but lose the Hml expression during their maturation (Mukherjee et al., 2011; Vanha-Aho et al., 2015). To examine whether modulating *PpV* in Hml^+^ cells has any impact on crystal cells, we used *hml-gal4* to drive *PpV RNAi* expression and assessed the number of crystal cells by Hnt staining. This manipulation caused a tremendous increase in the number of Hnt^+^ crystal cells compared to the *hml>gfp* control (Figure 5A–C), extending the impact of PpV on crystal cells to the Hml^+^ precursor stage.

Plasmatocytes are also developed from Hml^+^ precursors. Actually, 95% of Hml^+^ precursors eventually become plasmatocytes that retain stable Hml expression throughout the lineage. We then used the P1 marker to evaluate the changes of mature plasmatocytes upon *PpV* knockdown driven by *hml-gal4*. The P1^+^ plasmatocyte population did not significantly change in these lymph glands (Figure 5D–F), indicating that PpV has little effect on the production of plasmatocytes from Hml^+^ precursors. This also implied that *PpV* knockdown induced excessive crystal cells not at the expense of plasmatocytes. Lamellocytes are rarely observed under normal conditions but massively induced upon injury or parasitic wasp stress (Morin-Poulard et al., 2021; Evans et al., 2022). Using the L1 antibody, we demonstrated that knocking down *PpV* expression was unable to provoke the differentiation of lamellocytes (Figure 5G–H′).

Between the MZ (blood progenitors) and the CZ (plasmatocytes and crystal cells), intermediate progenitors co-expressing the MZ marker *dome* and the CZ marker *hml* reside (Blanco-Obregon et al., 2020; Rodrigues et al., 2021; Spratford et al., 2021). To examine whether blood progenitors (Dome^+^) and intermediate progenitors (Dome^+^Hml^+^) contribute to the regulation of crystal cells by PpV, we employed the *dome-gal4* driver to express *PpV RNAi*. However, *PpV* knockdown by *dome-gal4* did not cause an expansion of the crystal cell population (Figure 5I–K), suggesting that blood progenitors and intermediate progenitors were not involved in the PpV regulation of crystal cells.

Altogether, these results indicated that the increased crystal cells upon *PpV* depletion result from Hml^+^ crystal cell precursors, which initiate the differentiation from progenitors toward the crystal cell fate (Figure 5L).

### PpV regulates crystal cell survival in a Notch pathway-dependent manner

**Figure 6:**
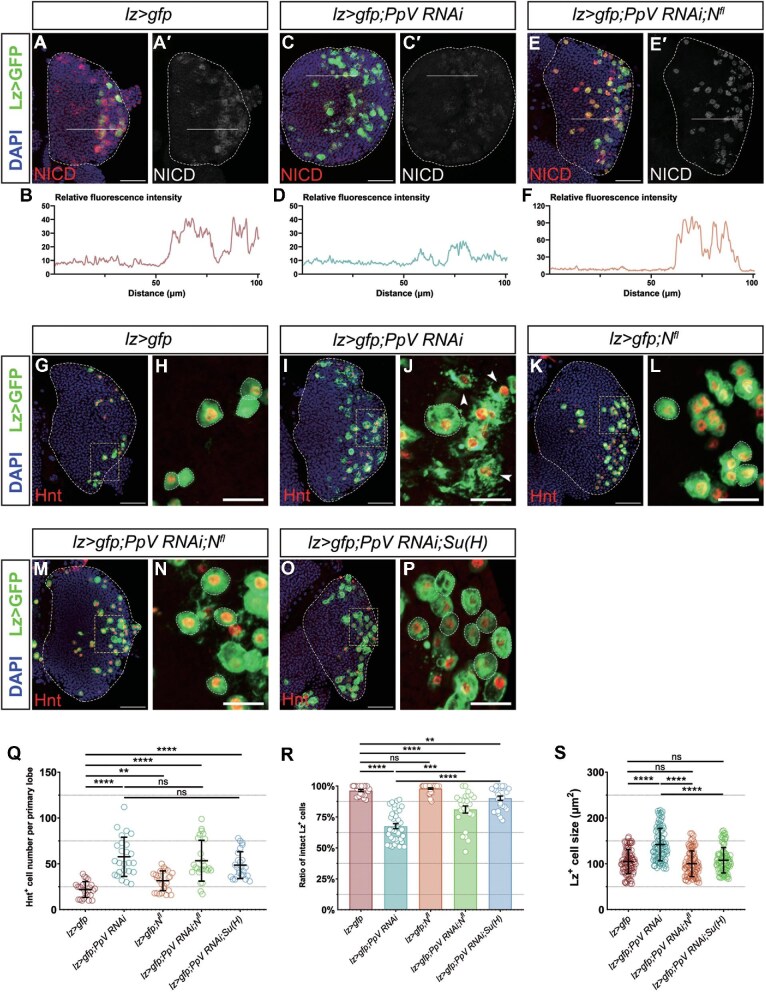
PpV regulates crystal cell maintenance but not cell number through Notch signaling. (**A**–**F**) NICD staining (red) of crystal cells (Lz>GFP) in the 3rd instar lymph gland. NICD staining (red) highly accumulates in crystal cells compared with the rest of the *lz>gfp* lymph gland, which is significantly reduced upon silencing *PpV* with RNAi. Additional full-length *Notch* (*N^fl^*) expression in *lz>gfp;PpV RNAi* lymph glands recovers the NICD expression level (red) in crystal cells. Panels **B, D**, and **F** demonstrate the relative fluorescence intensity of NICD at white solid lines in **A′, C′**, and **E′**, respectively. The horizontal axis represents the trend of white solid lines from the MZ (left) to the CZ (right). Scale bar, 50 μm. (**G**–**P**) Hnt (red) marks the nuclei of crystal cells (Lz>GFP) in lymph glands. Compared with the *lz>gfp* control, crystal cells in *lz>gfp;PpV RNAi* lymph gland increase in number and are ruptured, while crystal cells in *lz>gfp;N^fl^* as a positive control increase in number but do not show a rupture phenotype. Additional expression of *N^fl^* or *Su(H)* on the background of *lz>gfp;PpV RNAi* rescues the rupture phenotype but does not decrease the number of crystal cells. Panels **H, J, L, N**, and **P** are the 4× enlarged view of white-dashed boxes in **G, I, K, M**, and **O**, respectively. Arrows in **J** indicate ruptured crystal cells. (**Q**) The number of Hnt^+^ crystal cells per primary lobe for genotypes shown. Error bars indicate SD; *n* = 25. (**R**) The proportion of intact Lz^+^ crystal cells for genotypes shown. Error bars indicate SEM; *n* = 26, 36, 36, 25, and 27. (**S**) Quantification of the Lz^+^ crystal cell size for genotypes shown. Error bars indicate SD; *n* = 90. DAPI (blue) marks nuclei. Scale bar, 50 μm, except 20 μm for **H, J, L, N**, and **P**. **P < 0.01, ***P < 0.001, ****P < 0.0001; ns, no significance.

It has been known that Notch signaling is closely related to crystal cell fate determination and survival. Starting from the MZ, the Notch pathway allocates the cell fate of blood progenitors toward crystal cells and activates the transcriptional factor Lz to maintain the established developmental routine (Lebestky et al., 2003; Terriente-Felix et al., 2013; Blanco-Obregon et al., 2020). Then, non-canonical Notch signaling, which is independent of the ligand Serrate, facilitates the maintenance and survival of mature crystal cells (Mukherjee et al., 2011). We used a specific antibody to detect the Notch signaling effector, Notch intercellular domain (NICD), in lymph glands. Consistent with previous reports, NICD was located particularly in crystal cells, overlapping with Lz>GFP (Figure 6A and B). However, the expression level of NICD in crystal cells significantly decreased upon *PpV* depletion, suggesting that PpV could regulate crystal cells through Notch signaling (Figure 6C and D).

We therefore examined whether additional Notch could rescue the phenotypes caused by *PpV RNAi*. When co-expressing full-length *Notch* (*N^fl^* in short) with *PpV RNAi* driven by *lz-gal4*, the expression level of NICD was retrieved (Figure 6E and F). Meanwhile, the rupture of crystal cells was appreciably alleviated (Figure 6G–N and R), the Lz^+^ crystal cell size was restored to the *lz>gfp* control level (Figure 6S), but the increased number of crystal cells in the lymph gland could not be reduced to the *lz>gfp* control level (Figure 6Q). Notably, the number of crystal cells increased when *N^fl^* was expressed alone (Figure 6K and L), consistent with previous reports. Similarly, overexpression of the transcriptional activator of the Notch signaling pathway, Suppressor of Hairless [Su(H)], also rescued the rupture and oversize phenotypes of *PpV RNAi* (Figure 6O–S). These results strongly supported that PpV functions through Notch signaling to modulate crystal cell survival but not crystal cell number.

### PpV depletion activates crystal cell proliferation

**Figure 7:**
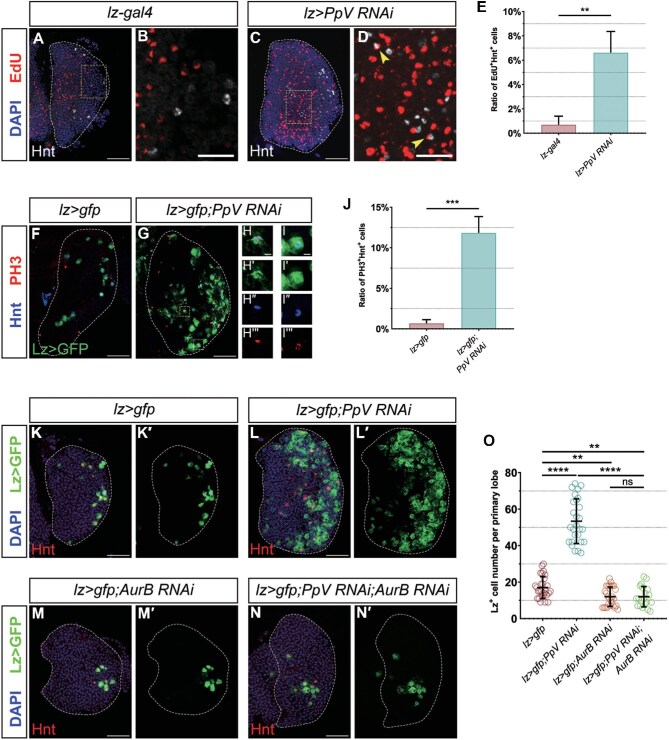
*PpV RNAi* promotes crystal cell proliferation. (**A**–**D**) Hnt^+^ crystal cells (white) barely overlap with EdU (red) in the *lz-gal4* control but co-localize with EdU (yellow arrows) upon *PpV* depletion. Panels **B** and **D** are the 4× enlarged view of white-dashed boxes in **A** and **C**, respectively. (**E**) The ratio of EdU^+^Hnt^+^ crystal cells for genotypes shown. Error bars indicate SEM; *n* = 13 and 14. (**F**–**I′′′**) Hnt^+^ (blue) crystal cells in primary lobes overlap with PH3 staining (red) upon *PpV* depletion. Panels **H**–**I′′′** are the 3× enlarged view of the white-dashed box in **G**. (**J**) The ratio of PH3^+^Hnt^+^ crystal cells for genotypes shown. Error bars indicate SEM; *n* = 7 and 11. (**K**–**N′**) Compared with the *lz>gfp* control, crystal cells (Lz>GFP) in *lz>gfp;PpV RNAi* lymph glands increase in number. Expressing *AurB RNAi*, either alone or on the background of *lz>gfp;PpV RNAi*, is able to decrease the number of crystal cells in the primary lobe. (**O**) The number of Lz^+^ crystal cells for genotypes shown. Error bars indicate SD; *n* = 28, 27, 27, and 20. DAPI marks nuclei. Scale bar, 50 μm, except 5 μm for **H**–**I′′′** or 20 μm for **B** and **D**. **P < 0.01, ***P < 0.001, ****P < 0.0001; ns, no significance.

To assess the proliferation rate of crystal cells in the 3rd instar lymph gland, we performed the EdU incorporation assay. Within the whole primary lobe, the EdU^+^ cells tended to be located in the MZ (Figure 7A). Then, we characterized crystal cells with Hnt staining. In *lz-gal4* control lymph glands, Hnt^+^ crystal cells were almost irrelevant to EdU signaling, while in *lz>PpV RNAi* lymph glands, Hnt^+^ crystal cells showed a mild degree of EdU incorporation (Figure 7A–E), suggesting that these crystal cells might enter mitosis upon *PpV* knockdown.

EdU has been reported to also mark endo-replicated cells. Since crystal cells may undergo endoreplication (Terriente-Felix et al., 2013), we used an antibody against phosphorylated histone H3 (PH3) to double-check whether crystal cells exhibit mitotic activities. Under normal conditions, very few PH3^+^ blood cells were observed in late 3rd instar primary lobes (Figure 7F), consistent with the inactive mitosis in the lymph gland at this stage as previously reported (Kapoor et al., 2022). Besides, Hnt^+^ crystal cells were almost irrelevant to PH3 staining (Figure 7F). However, when *PpV* was silenced by RNAi, ∼10% of Hnt ^+^ crystal cells, either intact or ruptured, were found showing PH3 staining (Figure 7G–J), indicating that more mitosis occurred in crystal cells in the *PpV RNAi* lymph glands.

Furthermore, we specifically silenced *Aurora B* (*AurB*), which encodes a serine–threonine kinase that promotes mitosis (Giet and Glover, 2001; Spratford et al., 2021), in crystal cells by RNAi. Compared to the *lz>gfp* control (Figure 7K and K′), the number of crystal cells in *lz>gfp;AurB RNAi* lymph glands only slightly decreased (Figure 7M and M′), suggesting that crystal cells usually do not undergo the cell cycle. When *AurB RNAi* was co-expressed with *lz>gfp;PpV RNAi*, the crystal cell number in lymph glands was restricted to a very low level close to that in *lz>gfp;AurB RNAi* lymph glands (Figure 7L–O). These data confirmed that the enhanced crystal cell proliferation is responsible for the increase in cell number upon *PpV* depletion.

### Overexpressing PpV suppresses the rupture of crystal cells in the lymph gland upon injury

**Figure 8:**
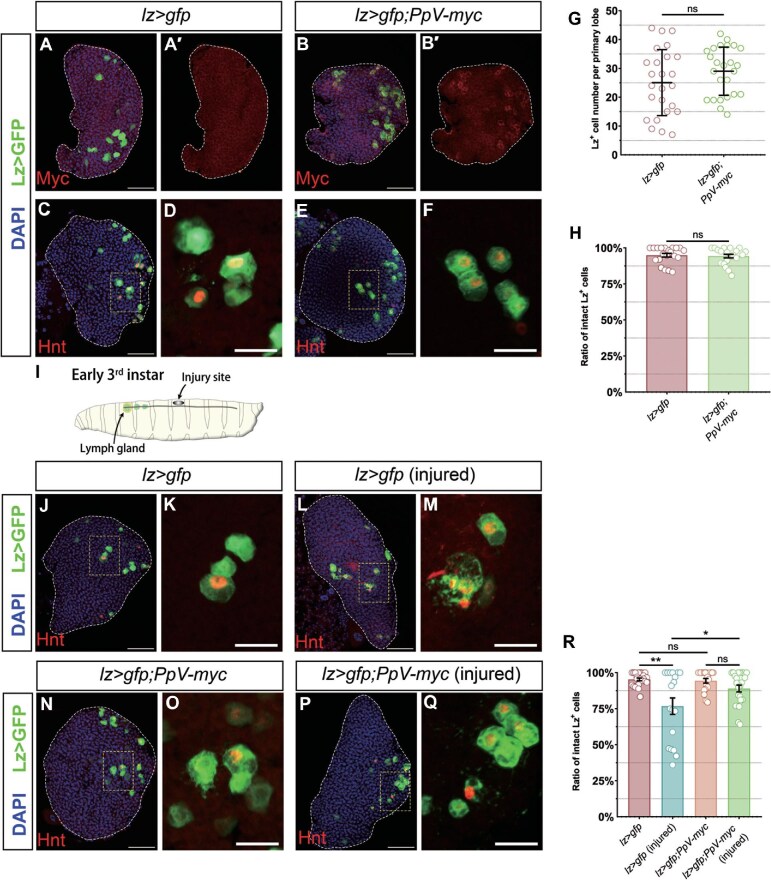
Overexpression of *PpV* prevents crystal cell rupture in the lymph gland upon injury. (**A**–**B′**) Myc staining (red) in *lz>gfp* and *lz>gfp;PpV-myc* primary lobes verifies the expression of exogenous *PpV*. (**C**–**F**) Hnt^+^ (red) crystal cells in *lz>gfp* and *lz>gfp;PpV RNAi* primary lobes do not exhibit significant differences in number and integrity. Panels **D** and **F** are the 4× enlarged view of white-dashed boxes in **C** and **E**, respectively. (**G**) The number of Lz^+^ crystal cells per primary lobe for genotypes shown. Error bars indicate SD; *n* = 25. (**H**) The proportion of intact Lz^+^ crystal cells for genotypes shown. Error bars indicate SEM; *n* = 21. (**I**) Schematic diagram of the injury site. (**J**–**Q**) Crystal cells (Lz>GFP) in *lz>gfp* larvae are partially ruptured upon injury. When *PpV-myc* is overexpressed, the injury-induced crystal cell rupture phenotype is diminished. Hnt (red) marks the nuclei of crystal cells. Panels **K, M, O**, and **Q** are the 4× enlarged view of white-dashed boxes in **J, L, N**, and **P**, respectively. (**R**) The proportion of intact Lz^+^ crystal cells for genotypes shown. Error bars indicate SEM; *n* = 22, 18, 22, and 23. DAPI marks nuclei. Scale bar, 50 μm, except 20 μm for **D, F, K, M, O**, and **Q**. *P < 0.05, **P < 0.01; ns, no significance.

Finally, we examined changes in crystal cells upon overexpressing *PpV* by using *lz-gal4* to drive *PpV-myc* expression (*lz>gfp;PpV-myc*). Staining with an antibody against Myc proved exogenous expression of PpV (Figure 8A–B′). However, *PpV-myc* did not alter the crystal cell population in the primary lobes or cause the rupture phenotype under normal conditions (Figure 8C–H).

Crystal cells can release cytosolic content through rupture to help wound healing during injury (Cattenoz et al., 2020; Dziedziech and Theopold, 2022). Previous studies reported that circulating crystal cells could be recruited to the wound site and function immediately to respond toward injury (Galko and Krasnow, 2004; Bidla et al., 2007; Binggeli et al., 2014). However, the influence of injury on lymph gland crystal cells remains largely unknown. Thus, we pricked early 3rd instar larvae (96 h after egg laying) with a needle and examined the status of crystal cells in the lymph gland (Figure 8I). After 24 h, lymph glands in the injured *lz>gfp* larvae contained considerable ruptured crystal cells that were triggered by the mechanical injury (Figure 8J–M and R), while fewer crystal cells were ruptured in the injured *lz>gfp;PpV-myc* larvae (Figure 8N–R), suggesting a role of PpV in protecting crystal cells from rupture during injury response, which is worthy of further exploration.

## Discussion

Here, we reported the expression pattern of PpV in the lymph gland and a novel function of PpV in *Drosophila* hematopoiesis. PpV is accumulated specifically in crystal cells of lymph glands and could be applied as a new and reliable indicator for crystal cells. The function studies revealed that PpV is required for maintaining the number and integrity of crystal cells under normal and injury conditions. In addition, PpV protein serves as the catalytic subunit of the PP6 holoenzyme, and Fmt protein is the regulatory subunit of the PP6 holoenzyme in *Drosophila*. Our data confirmed that *PpV RNAi* and *fmt RNAi* have the same effect on crystal cell number and integrity, suggesting that PP6 holoenzyme activity is essential in this context.

The proliferative status of crystal cells has rarely been observed under normal conditions (Sinenko et al., 2009). There could be certain limitations that restrict the extra proliferation of crystal cells and maintain them at a low number. Based on our study, PpV might serve as a limitation factor. Likewise, the number of nuclear divisions during embryogenesis under normal circumstances should be strictly controlled, while the embryos of *pph-6*-inactivated *Caenorhabditis elegans* or *PpV*-null mutant *Drosophila* often undergo extranuclear divisions (Afshar et al., 2016; Liu et al., 2019). Our study, as well as the previous reports, suggested that *PpV* is not only involved in mitosis but also a key factor in the precise control of the cell proliferative status under certain contexts requiring strict restriction on cell divisions.

Our analysis of single-cell transcriptomic data showed that the expression levels of *PpV* in both circulating crystal cells and lymph gland crystal cells significantly decrease upon injury or infestation, suggesting that low levels of PpV may contribute to wound healing or the removal of invaders. Consistently, our experimental data showed that *PpV* depletion induces crystal cell enlargement and even rupture, which triggers the release of PPO crystalline inclusions required for melanization. We also noticed that most swollen crystal cells contain huge crystalline inclusions, implying that the continued enlargement of the crystalline may be the cause of the swelling and eventual rupture of crystal cells. On the other hand, overexpression of *PpV* can inhibit the rupture of crystal cells to a certain extent. Therefore, we hypothesize that PpV normally functions to restrict the production of PPO, whereas under conditions of injury or infestation, cells reduce *PpV* expression levels to promote PPO production, the PPO crystals become larger and burst crystal cells, and the released PPO enzymes participate in melanization to promote wound healing and invader clearance.

PP6 holoenzyme has been linked with mitosis in yeast, *Drosophila*, and human cells (Bastians and Ponstingl, 1996; Goshima et al., 2003; Zeng et al., 2010; Liu et al., 2019) through Aurora A (AurA), an essential mitotic kinase as a well-established substrate of the PP6 complex (Liu et al., 2019; Ohama, 2019). Our results showed that the *PpV RNAi*-induced increase in crystal cell number can be diminished by knocking down *AurB*. In homology analysis, AurA and AurB share 51.9% identity at the level of amino acid sequences, and many critical residues are conserved between AurA and AurB (Liu et al., 2019). Thus, it is possible that AurB is also a substrate of PP6, though additional studies are needed to confirm this hypothesis.

PpV has been implicated in AMPK and JNK pathways in *Drosophila* (Yin et al., 2014; Ma et al., 2017; Chi et al., 2018). Here, we demonstrated that Notch is also associated with PpV. The Notch signaling pathway has been linked with the hematopoietic system at multiple levels. In mammals, Notch activation promotes the myeloid lineage differentiation of hemopoietic progenitor cells (Schroeder et al., 2003; Zhou et al., 2008). Similarly, the Notch pathway guides the differentiation and maturation of crystal cells in *Drosophila* (Duvic et al., 2002; Lebestky et al., 2003). However, our data indicated that the Notch pathway is not responsible for the increased number of crystal cells in the *PpV RNAi* lymph gland. Moreover, the ineffectiveness of *dome-gal4* implied that the functions of Notch in ‘distal progenitors’ for binary cell fate specification and in intermediate progenitors for inducing crystal cells are not applicable to this situation (Blanco-Obregon et al., 2020; Spratford et al., 2021). On the other hand, Serrate-independent Notch signaling is required in mature crystal cells to sustain their survival and integrity (Mukherjee et al., 2011). In our experiments, the exogenous Notch activation largely rescued the destabilization of crystal cells, suggesting that Notch signaling may play a role in cell integrity maintenance, because we also captured many enlarged crystal cells upon *PpV* depletion, which seems like a prelude to rupture. Further exploration of how PpV impacts the activity of Notch signaling will be pursued.

Crystal cells are nonphagocytic cells, mostly known for facilitating the wound healing process during injury and immune stress (Lanot et al., 2001). These cells are functionally closest to human platelets, although the latter are much smaller in size (Banerjee et al., 2019; Montague et al., 2020). In addition, crystal cells express the transcription factor Lz, which contains the RUNT domain and is the ortholog of Runx1 (also known as acute myeloid leukemia-1, AML1). *Runx1* is described as an early marker of mammal hematopoiesis and most reported in the chromosomal translocation associated with AML in humans (Mathey-Prevot and Perrimon, 1998; Dzierzak and Speck, 2008). Myeloid leukemia factor 1 (MLF1) is another identified target for human AML. In flies, MLF has been demonstrated to regulate crystal cell development by stabilizing Lz (Desplan et al., 2017). Also, the Notch signaling pathway has been extensively investigated in different kinds of mammalian blood cells, including hematopoietic stem cells (HSCs), hematopoietic progenitors, and mature blood cells (Blanco-Obregon et al., 2020). During the embryonic stage, Notch is reported to be necessary for the generation of HSCs in the AGM region (Bigas and Espinosa, 2012). Thus, crystal cells share developmental and functional homology with human blood cells in many ways. Our work showcased a novel function of PpV in regulating crystal cell status in *Drosophila*, which could shed light on investigating the possible function of PP6 in mammalian hematopoietic systems and innate immune response.

## Materials and methods

### Fly strains and husbandry

Fly strains *hml-gal4,UAS-gfp* (#30140), *lz-gal4,UAS-gfp* (#6314), and *UAS-fmt RNAi* (#35597) were obtained from Bloomington *Drosophila* Stock Center. Fly strains *UAS-gfp* (THJ0079), *UAS-AurB RNAi* (THU4045), *UAS-PpV RNAi* (TH03654.N), *tub-gal4* (TB00129), *w^1118^* (THJ0265), and *yw* (THJ0266) were obtained from Tsinghua Fly Center. The fly strain *UAS-PpV RNAi* (#101997) was obtained from Vienna *Drosophila* Resource Center. The fly strain *UAS-Su(H) ORF-3×HA* (F001922) was obtained from FlyORF. *UAS-PpV-myc* (a Myc tag was fused to the C-terminus of wild-type *ppv* coding region, which was subcloned into the pUAST vector) was made by standard P element-mediated transformation. The following fly lines were generously provided as indicated: *dome-gal4/FM7C* by Dr M. Crozatier (Université Paul Sabatier Toulouse III, France), *PPO45-lacZ* by Dr L. Waltzer (Université Paul Sabatier Toulouse III, France), and *UAS-N^fl^* by Dr Alan J. Zhu (Peking University, China). *w^1118^* was used as background control for PpV staining. All stocks were raised on corn meal-based *Drosophila* medium at 25°C.

### RNA extraction and sequencing

The 3rd instar larvae were collected and homogenized with a tissue grinder in TRIzol reagent. The smashed samples were left at room temperature for 5 min to ensure proper dissociation of nucleoprotein complexes, followed by centrifugation at 12000× *g* for 5 min. The supernatant was collected and transferred to the new RNase-free centrifuge tubes. Chloroform was added to the supernatant. The samples were mixed mildly and left at room temperature for 15 min. The upper aqueous phase containing the RNA was carefully collected and transferred to a new RNase-free tube. Isopropanol was added to the aqueous phase. The samples were mixed mildly and left at room temperature for 10 min, followed by centrifugation at 12000× *g* for 10 min at 4°C. The supernatant was discarded. The RNA was precipitated at the bottom of the tubes. Then, the RNA was washed with 75% ethanol and centrifuged at 12000× *g* for 10 min at 4°C again. The precipitated RNA was left at room temperature for air-drying. RNase-free water was added to each sample. NanoDrop 2000 (Thermo Scientific) was used to test the concentration of total RNA.

For RNA-seq, Agilent 2100 bioanalyzer (Agilent Technologies) was first used to check the integrity of total RNA. The mRNA with polyA tails was enriched with Oligo(dT) magnetic beads, fragmented into 250–300 bp, and reverse-transcribed to double-stranded cDNA (ds-cDNA). Indexed Illumina adapters were ligated to ds-cDNA. Next, ds-cDNA of ∼200 bp was screened using AMPure XP beads and amplified by polymerase chain reaction, followed by a screen with AMPure XP beads again. Then, the cDNA libraries were established. Qubit 2.0 (Invitrogen) was used for quantification and Agilent 2100 bioanalyzer was used to check the cDNA library insert size. The sequencing was performed at Novogene Inc. using an Illumina PE150 (pair end 150 bp) platform.

### Bioinformatic analysis of RNA-seq data

The raw RNA-seq data was first filtered to obtain clean reads and then analyzed by error rate and GC content. Subsequently, Hisat2 was used for mapping the reads to the reference genome (FlyBase dmel-r6.15 genome). StringTie was used to assemble and quantify the transcripts. Fragments per kilobase of million mapping reads (FPKM) were used to determine the abundance of transcripts. edgeR was used to analyze the significance of expression differences of transcripts/genes. Three to five biological replicates were established to select DEGs with a *P*-value <0.05. The volcano plot was used to visualize the distribution of −log_10_(*P*-value) and log_2_(fold change) values of DEGs. The heatmap was used to show the log_2_FPKM values of differentially expressed hemocyte marker genes. GOseq was used to perform GO enrichment analysis for DEGs. For dot plots, the mean level of *PpV* expression and the percentage of cells that show *PpV* expression were analyzed for each cluster using the R package (ggplot2).

### Antibodies and immunostaining of lymph glands

The following antibodies were used: mouse P1 antibody (1:200) and mouse L1 antibody as a gift from Dr I. Ando (Biological Research Center, Szeged, Hungary), mouse anti-NICD (1:200) and mouse anti-Hindsight (Hnt, 1:100) obtained from Development Studies Hybridoma Bank, rabbit anti-PpV (1:400) generated by GenScript, rabbit anti-PH3 (1:100) obtained from Affinity Biosciences, mouse anti-β-gal (1:2000) obtained from Cell Signaling Technology, and Alexa Fluor-conjugated goat anti-mouse/rabbit IgG (1:200) and phalloidin-555 (1:1000) obtained from Thermo Fisher Scientific.

For immunostaining of lymph glands, late 3rd instar larvae were inverted in phosphate-buffered saline (PBS) and fixed in 4% paraformaldehyde (PFA) for 30 min at room temperature. The samples were washed three times in PBS with 0.1% Triton-X (PBST), each for 5 min, and blocked in 0.2% bovine serum albumin (BSA)/PBST for 1 h. Then, these samples were incubated with the primary antibody at 4°C overnight, washed three times in PBST, and incubated with the corresponding secondary antibody at room temperature for 1 h. Finally, after further PBST wash (three times, 5 min each), lymph glands were dissected from the samples and mounted in a VECTASHIELD mounting medium with DAPI for labeling the nuclei (Vector Laboratories).

All circulating blood cells from late 3rd instar larvae were collected onto coverslips and subjected to immunostaining with the same protocol as lymph glands.

### PPO activation

The crystal cells in circulation or attached to the body wall were visualized through PPO activation. Specifically, late 3rd instar larvae were collected in centrifuge tubes with PBS and incubated at 60°C for 10 min.

### EdU labeling

EdU labeling was performed using the Life Biotech Future EdU Cell Proliferation kit. First, the larvae were inverted in PBS, incubated in PBS with EdU (1:1000) for 35 min at room temperature, and then fixed in 4% PFA for 30 min at room temperature. The samples were washed four times in 0.3% PBST, each for 10 min. Next, if a co-staining with antibody was required, the samples were blocked and incubated with primary and secondary antibodies before the following steps; otherwise, the samples were directly immersed in the supporting EdU staining solution as per the manufacturer's instruction for 30 min at room temperature. These samples were washed sequentially with 0.3% PBST twice and PBS once, each for 10 min. The lymph glands were finally dissected from the samples and mounted in a VECTASHIELD mounting medium with DAPI (Vector Laboratories).

### Puncture injury

Early 3rd instar larvae (96 h after egg laying) were randomly picked and pricked with a thin needle (∼200 μm in diameter). Then, these injured larvae were placed into 35-mm culture dishes with corn meal-based *Drosophila* medium at 25°C. After 24 h, the larvae were picked out from the medium for immunostaining of lymph glands.

### Image acquisition and analysis

Slides were imaged using a fluorescence microscope (Zeiss Imager Z2) and confocal microscope (Leica TCS SP8). Larvae were imaged using a stereo microscope (Nikon SMZ1270).

All the results were concluded from at least three separate experiments to ensure reproducibility. For quantification analysis, at least eight randomly picked lymph glands were analyzed for each genotype. The primary lobe size was calculated using Adobe Photoshop and ImageJ according to the DAPI nuclei staining. To characterize the size of single crystal cells, the Lz^+^ cells with clear boundaries were chosen and measured using ImageJ. The number of crystal cells in each primary lobe was counted manually with Lz>GFP or Hnt labeling. The crystal cells in circulation/attached to the body wall were counted manually. The total P1^+^ area representing plasmatocytes was measured by ImageJ. The number of EdU^+^ cells was counted using ImageJ.

Statistical analyses were performed using GraphPad Prism 8.0, and the statistical significance was determined using an unpaired *t*-test. Error bars indicate standard deviation (SD) or standard error of the mean (SEM). Statistical significance was established as *P < 0.05, **P < 0.01, ***P < 0.001, or ****P < 0.0001; ‘ns’ indicates no significance.

## Supplementary Material

mjae028_Supplemental_File

## Notes

**Author contributions:** Y.S., L.Z., and W.L. designed the study; W.L., F. Zhang, F. Zhao, and Y.F. performed experiments and data analyses; W.L. wrote the manuscript; Y.S. improved the writing.
