# Supplementary material for: Dual role of PpV in Drosophila crystal cell proliferation and survival
Source: J Mol Cell Biol. 2024 Jul 31;16(9):mjae028. doi: 10.1093/jmcb/mjae028 (PMC11927399; doi:10.1093/jmcb/mjae028)
Supplement: mjae028_Supplemental_File [file mjae028_supplemental_file.pdf]

Supplementary material

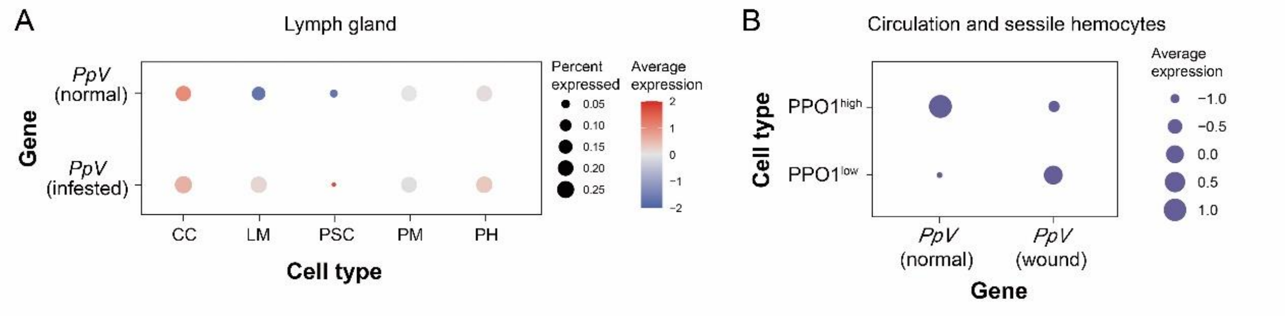

Supplementary Figure S1 PpV expression in crystal cells by analyzing the published single-cell transcriptomic data. (**A**) Analysis of single-cell RNA-seq data of *Drosophila* lymph glands (Cho et al., 2020) revealed a high expression level of *PpV* in crystal cells (CC), but a very low level in lamellocytes (LM), posterior signaling center (PSC), plasmatocytes (PM), and progenitor cells (prohemocytes, PH) under normal condition. Upon infestation, *PpV* expression level in crystal cells is mildly reduced. The mean level of *PpV* expression is represented by the dot color, and the percentage of *PpV*-expressing cells is indicated by the dot size. (**B**) Analysis of single-cell RNA-seq data of circulating blood cells in *Drosophila* larvae (Tattikota et al., 2020) indicated that *PpV* highly expresses in the cell population with high PPO1 expression ($PPO1^{high}$) under normal conditions. Upon wound, *PpV* expression level in this population is dramatically reduced. Dot size signifies the average expression level of *PpV*.

References

Cho, B., Yoon, S.H., Lee, D., et al. (2020). Single-cell transcriptome maps of myeloid blood cell lineages in Drosophila. Nat. Commun. *11*, 4483.

Tattikota, S.G., Cho, B., Liu, Y., et al. (2020). A single-cell survey of Drosophila blood. eLife *9*, e54818.

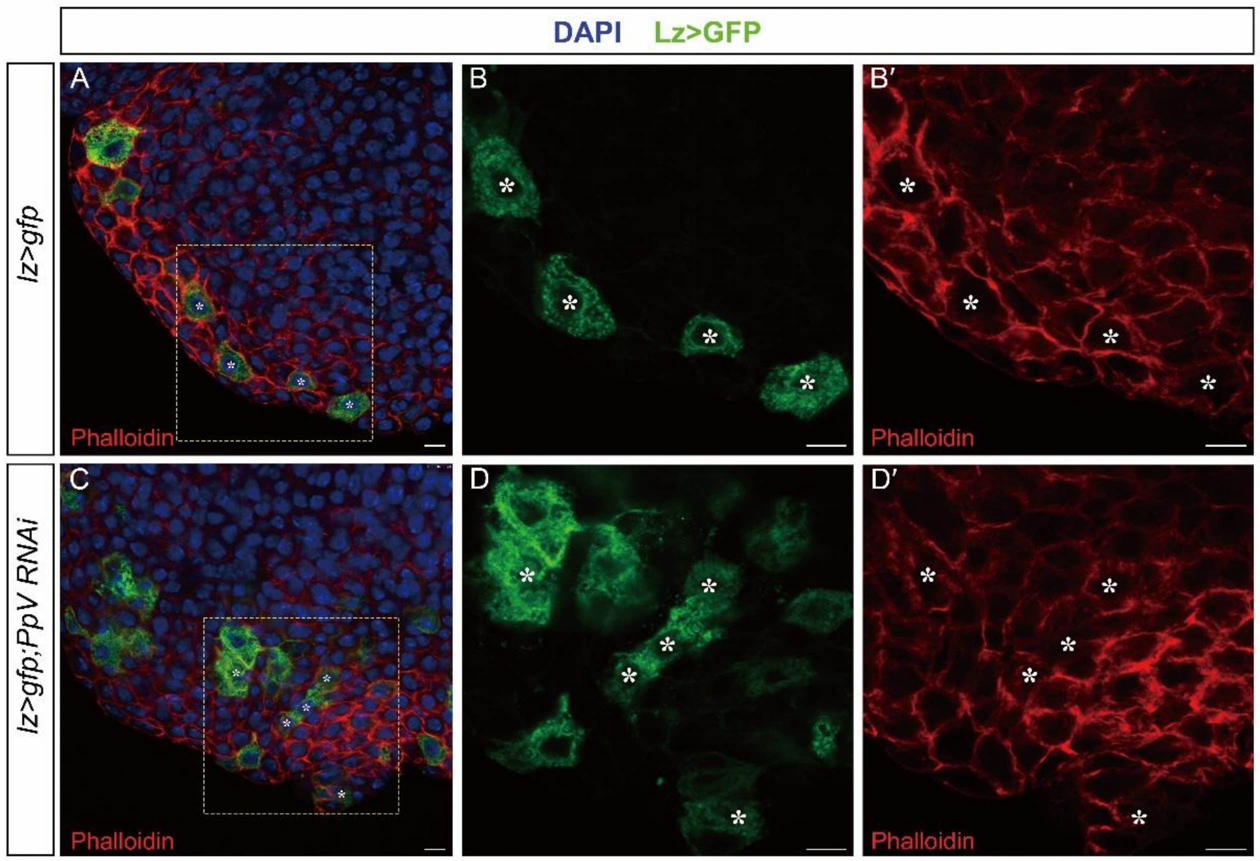

Supplementary Figure S2 PpV regulates the integrity of crystal cells in lymph glands. The intact cytoskeleton labeled by Phalloidin (red) occurs in *lz>gfp* control lymph glands (**A**–**B′**), while cytoskeleton of rupture crystal cells in the *lz>gfp;PpV RNAi* lymph glands (**C**–**D′**) is fragmentary. Panels **B**–**B′** and **D**–**D′** are the 2× enlarged views of yellow-dashed boxes in panels **A** and **C**, respectively. White arrows indicate crystal cells with disrupted cytoskeletons. GFP driven by *lz-gal4* (Lz>GFP) marks the crystal cells. DAPI (blue) marks nuclei. Scale bar, 6 μm.
